# A Comparison between Three Different Automated Total 25-hydroxyvitamin D Immunoassay Methods and Ultra Performance Liquid Chromatography

**DOI:** 10.31557/APJCP.2020.21.4.1039

**Published:** 2020-04

**Authors:** Hala Reda, Sonya Soliman, Hany Girguis, Mohamed Nagy, Yousra Mahmoud, Nouran Yasser

**Affiliations:** 1 *Clinical Pathology Department, NCI, *; 2 *Clinical Pathology Department, *; 3 *Personalized Medication Management Unit, Pharmacy, *; 4 *Clinical Research Specialist, Clinical Research Department, Epidemiology and Bio-statistics Unit, CCHE, Cairo, Egypt. *

**Keywords:** Vitamin D, vitamin D method comparison, Vitamin D standardization, 25-Hydroxy vitamin D

## Abstract

**Objective:**

Vitamin D is a fat soluble vitamin responsible for calcium metabolism and more recently discovered effects. This led to an increase in requests for vitamin D test by clinicians. New automated assays have been introduced for 25-hydroxyvitamin D measurement.

**Methods:**

Results from these new method have to be related to a Standard method to obtain best results for practical usage. In our study, one hundred venous blood samples were analyzed for 25-OH vitamin D on three immunological methods in our lab and correlated with ultra-performance liquid chromatography (UPLC) method as a reference method.

**Results:**

Statistically analysis of results obtained for correlations between the 3 methods against the reference UPLC was done by Spearman’s Correlation. It showed positive correlation in all methods with significant p value < 0.001. Differences and biases between methods were evaluated using a Bland-Altman plot and Cohen’s Kappa agreement. Best agreement was found in Cobas 6000 followed by the Access2 then comes Architect.

**Conclusions:**

All immunoassays can be used in routine 25(OH) D measurements, still some methods are better than others. A clinical laboratory must at least be aware of its method to avoid misinterpretation of results.

## Introduction

Vitamin D is a group of fat-soluble secosteroids responsible for increasing intestinal absorption of calcium, magnesium, and phosphate, and multiple other biological effects. The major natural source of the vitamin is synthesis of cholecalciferol in the skin from cholesterol through a chemical reaction that is dependent on sun exposure (specifically UVB radiation) (Holick, 2004). 

Vitamin D from the diet or skin synthesis is biologically inactive; enzymatic conversion (hydroxylation) in the liver and kidney is required for activation. As vitamin D can be synthesized in adequate amounts by most mammals exposed to sufficient sunlight, it is not an essential dietary factor, and so not technically a vitamin. Instead, it could be considered a hormone, with activation of the vitamin D pro-hormone resulting in the active form, calcitriol, which then produces effects via a nuclear receptor in multiple locations (Norman, 2008). Cholecalciferol is converted in the liver to calcifediol (25-hydroxycholecalciferol); ergocalciferol is converted to 25-hydroxyergocalciferol. These two vitamin D metabolites (called 25-hydroxyvitamin D or 25(OH) D) are measured in serum to determine a person’s vitamin D status. 

A diet deficient in vitamin D in conjunction with inadequate sun exposure causes osteomalacia (or rickets when it occurs in children). However, vitamin D deficiency has become a worldwide problem in the elderly and remains common in children and adults (Holick, 2007). 

Previous studies have reported that vitamin D is not only required for bone health, but also plays a role in autoimmune diseases, obesity, type 2 diabetes mellitus, metabolic syndromes, cardiovascular diseases, and certain types of cancers (Ganji et al., 2011). These reports have recently led to an increase in requests for vitamin D test by clinicians (Vanlint, 2013).

As a consequence, a number of new automated assays have been introduced for 25-hydroxyvitamin D measurement. However, there is a little consensus on which assay method should be used to measure its concentration, and there are serious concerns regarding the reliability of its measurement (Lai et al., 2010).

Several specifications should be considered when selecting a vitamin D assay, including total 25(OH)D measurement (the sum of 25(OH)D2 and 25(OH)D3), accuracy, reproducibility, turn-around time, inter-assay comparability, and cost- effectiveness (Farrell and Herrmann, 2013). Defects in automated chemiluminescent immunoassays (CLIA) are due to poor antibody specificity, with cross-reactivity to other vitamin D metabolites, incomplete extraction of the 25(OH) D from the binding protein, and matrix substances such as lipids (Kocak et al., 2015). 

Ultra performance liquid chromatography (UPLC) is considered a candidate reference method for accurate quantification of 25 (OH) D. However, HLPC technique has several drawbacks compared to automated immunoassays, including higher complexity, longer turnaround time and the need for skilled personnel, which make it virtually unavailable to some laboratories. Therefore, automated assays are typically regarded as the best choice for a number of laboratory services, provided that these methods display satisfactory analytical performance and optimal agreement with the reference techniques (Madenci et al., 2017). 

Thus we need to compare results of various automated immunoassays with those of (UPLC) as a reference method of assay, to point out the method that gives most matching results in terms of accuracy, precision, linearity, and agreement to be recommended for practical usage.

## Materials and Methods

*Study design and subjects*

This is an analytical method evaluation study. One hundred venous blood samples from adult (66) females and (34) adult males were randomly selected from our hospital staff. They have no pathologic laboratory results and taking no medications. 

*Blood sampling*

Two blood samples were taken from one puncture in the antecubital vein into 2 Vacutainer Serum Gel Separating Tubes. All tubes used in the study have the same Lot Number. Blood samples were centrifuged at 3,000 x g for 10 minutes. After exclusion of hemolytic, lipemic, icteric serum samples and those with insufficient volume from the study, the number of samples included in our study was 92 samples only (61) females and (31) adult males.

Serum was divided into four aliquots and stored at (–20^o^C) for a maximum of 30 days and analyzed in batches on the four systems. Analytical performance of newly developed 25 (OH) D assays was assessed on three platforms in our Chemistry lab.: Abbott Architect I 1000SR (Abbott Laboratories, Wiesbaden, Germany) by chemiluminescent microparticle immunoassay using Abbott Architect 25-OH vitamin D assay reagent and Roche Cobas 6000’s module e601 (Roche Diagnostics GmbH, Mannheim, Germany) by electrochemiluminescence immunoassay using Roche Cobas vitamin D total assay reagent. The third is the Bechman Coulter Access2 25 (OH) D by Access 2 kits competitive chemiluminescence enzyme immune assay. Then the fourth aliquot was analyzed by UPLC method by ClinRep RECIPE. This is considered as a reference method, with which all the former methods will be accessed. 

*Methods*

Samples were processed in a single batch on each analyzer according to the manufacturer’s instructions. Calibration curves were constructed using calibrators provided each kit of each analyzer. The fourth aliquot was stored at (-20°C). After one week of storage, collected aliquots were analyzed for 25 (OH) D by UPLC. Before analysis, frozen samples were thawed and single processing was conducted.

Ultra-Performance Liquid Chromatography (UPLC) has substantially increased analytical performance in comparison with conventional high performance liquid chromatography systems (HPLC) or even liquid chromatography tandem mass spectrometry (LC-MS/MS). Significant chromatographic advantages, based upon innovative column materials as well as more powerful injection systems, pumps and detectors, sharper peaks with considerably reduced retention times are obtained. Thus, UPLC may handle higher sample throughput with better quality of the analytical results at the same time.

The UPLC assay method was adapted from a method described by the manufacturer.

RECIPE Vitamin D kit measures 25-OHVitamin D2/D3 allows a fast and reliable determination of both analytes with an injection interval of only 2 minutes. Plasma or serum can be used for the analysis. But for better comparison only serum samples are used. This is considered a good quality and reliable certified analytical method. 

System check, calibration and internal quality control, all were used from reagents provided by the kit. The ClinCal Serum Calibrator is traceable to a certified reference material (NIST standard SRM 972). Also RECIPE was certified according to ISO 9001 and ISO 13485. Samples were prepared using a protein precipitation protocol according to the kit. Quantification was performed by linear regression of peak area ratios against the calibrator concentrations. UPLC is characterized by Isocratic pump, with flow rate is 0.75 ml/min, and injection volume 5 UL, and injection interval 2 min, with UV-Detector 264 nm and Column heater 40°C.

Access 2 the reportable measuring range of the assay is 2 to 166 ng/mL (4.9 to 416 nmol/L). 

Cobas e601 the measuring range is 3 to 70 ng/mL (7.5 - 175.0 nmol/L). 

Architect I 1000SR the measuring range is 5 to 160 ng/mL (12.8 - 400.0 nmol/L) 

*Statistical analysis*

The 25(OH) D results obtained by UPLC were used as the reference for method comparison studies. Results reading outside the measuring ranges of the immunoassay methods were omitted from statistical evaluation. Concentrations of 25(OH) D were given in ng/ml. The data was described using medians and Interquartile ranges for the numerical results of the techniques, Frequency and percentages for the categorical interpretations of the results. The data was checked for normality by visual histogram plotting, and Kolmogrov Smirnov test and were found to be not normally distributed. 

Correlations between the 3 methods against the reference UPLC was conducted by Spearman’s Correlation. Also, correlations between the 3 techniques against each other were performed. The results of all measurements were analyzed by Cohen’s Kappa agreement, and Bland & Altman plots. A P value < 0.05 indicates a significant deviation from linearity. 

Bland-Altman plot was used to assess differences and biases between methods. Bland and Altman recommend plotting differences against the average of the methods rather than against that of the reference method (Bland and Altman, 1995).

On the other hand Clinical and Laboratory Standards Institute (CLSI) recommends plotting differences against the reference method. Therefore, differences between values from comparative immunoassays and the reference method against the reference method value were displayed in the difference plots according to CLSI recommendations (Enko, 2015). 

The differences expressed as a percentage of the reference method value were plotted to illustrate whether the difference between the measurements made using the two methods was related to the magnitude of the measurement. Inter-rater agreement in assessment of vitamin D status between assays was analyzed using kappa (κ) analysis (McHugh, 2012). Statistical analysis was performed using PASS’15 software (CCHE 57357, Egypt).

## Results

25 (OH) D results obtained by UPLC were used as the reference and the range of the samples was (5.0 to 42.0) ng/ml. Samples giving results inside the reportable range of UPLC were 52 samples, and they could be evaluated in statistical analysis. Also, samples which have Values out of the measuring range of the immunological methods, were excluded from statistical analyses. 

Overall, 87 complete cases examined could be evaluated for Architect I 1000SR, 90 complete cases could be evaluated for Roche Cobas 6000’s module e601 and 90 complete cases could be evaluated for Bechman Coulter Access2 statistical analysis.

All of the 52 samples have results from the UPLS had results on all other 3 immunological methods for statistical analysis. We found that all data from the three different assay methods were not distributed normally based on Kolmogorov-Smirnov tests (P < 0.001).

The examined ranges of the 25(OH)D concentrations were 5 – 42 ng/ml as measured by UPLC, 3.4 - 65.9 ng/ml as measured by Cobas e601, 2.9- 41.8 ng/ml as measured by Architect I 1000SR and 2.9-43.4 ng/ml as measured by Access 2.

Spearman Correlations (r) was done to investigate correlation of each of the 3 methods with the standard UPLC. It was a positive correlation in the 3 methods with significant p value < 0.001 as shown in Table 1.

Upon doing the Spearman’s Correlation Coefficient (r) to investigate the correlation between the immunological tests with each other, also a significant positive correlation was seen as in Table 2.

*Differences and biases between methods were evaluated using a Bland-Altman plot*

**Figure 1:**
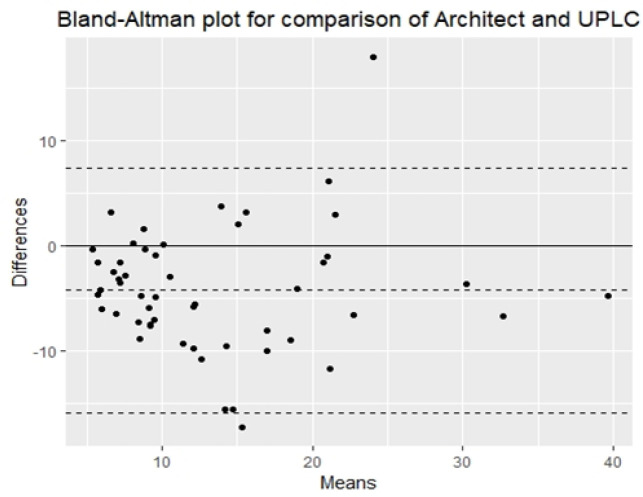
Comparison of the Abbott Architect with UPLC Method using Bland-Altman Analysis

For Architect versus UPLC the Bias = (-38.73%), standard deviation = 5.93 as in Figure 1.

**Figure 2:**
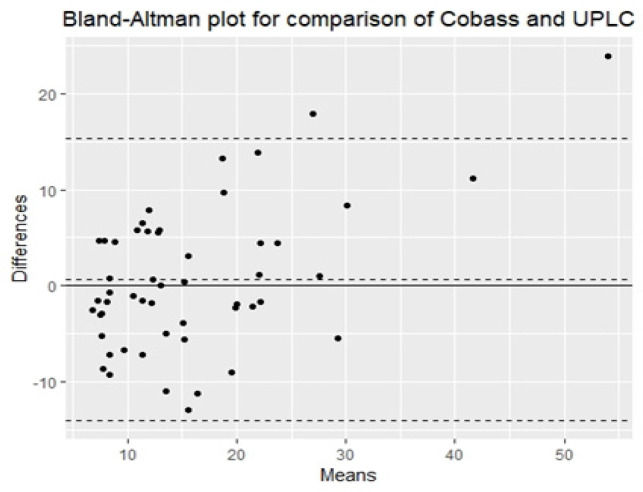
Comparison of the Cobas e601 with UPLC Using Bland-Altman Analysis

For Cobas e601 versus UPLC the Bias = (-4.22%), Standard Deviation = 7.49 as in Figure 2.

**Figure 3:**
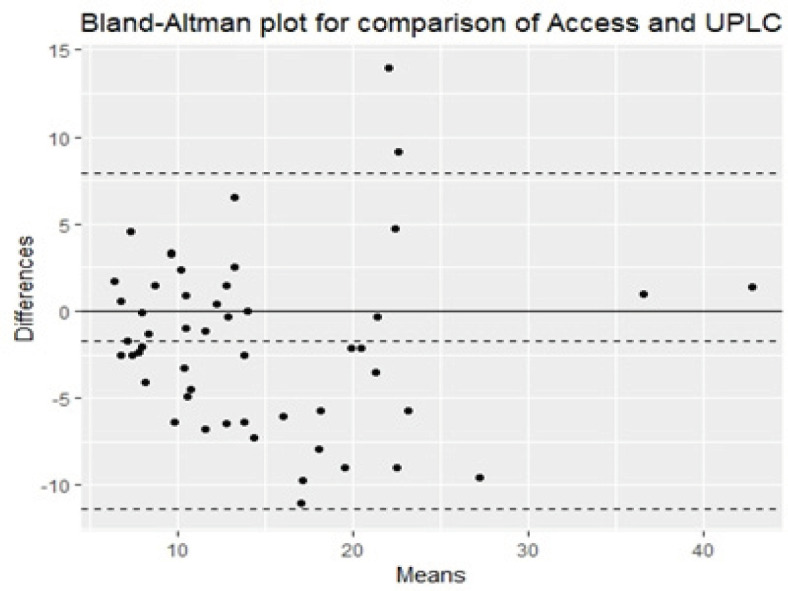
Comparison of the Access2 with UPLC using Bland-Altman Analysis

For Access2 versus UPLC, the Bias = (-12.34%), Standard Deviation = 4.93 as in Figure 3.

Also biases between methods were evaluated using a Bland-Altman plot on the samples that show deficiency of vitamin D (< 20 ng/ml) by the standard UPLC method.

*Bias from vitamin D deficient samples*

Architect – UPLC bias = -2.90 (-37.32%) StD = 5.50

Cobass – UPLC bias = 0.67 (-3.00%), stD = 6.74

Access – UPLC bias = -0.58 (-7.75%), stD = 4.53

Also, Kappa statistics was done to investigate agreement with the UPLS standard method: 

Based on the patient clinical status (k) Kappa statistic was used:

It showed the least agreement in case of Architect versus UPLC as shown in Table 3. For Cobas versus UPLC kappa statistic it made the best agreement as in Table 4. Also moderate agreement was seen in case of Access 2 versus UPLC as shown in Table 5.

**Table 1:** Correlation of each of the 3 Methods with the Standard UPLC

Architect vs UPLC n=52	Spearman's Correlation Coefficient ( r )	0.637
	*P*-value	<0.001
Cobass vs UPLC n=52	Spearman's Correlation Coefficient ( r )	0.580
	*P*-value	<0.001
Access vs UPLC n=52	Spearman's Correlation Coefficient ( r )	0.736
	*P*-value	<0.001

**Table 2:** Correlation of each of the 3 Methods with each other

Architect vs Cobass n=87	Spearman's Correlation Coefficient ( r )	0.772
	P-value	<0.001
Architect vs Access n=86	Spearman's Correlation Coefficient ( r )	0.882
	P-value	<0.001
Access vs Cobass n=89	Spearman's Correlation Coefficient ( r )	0.692
	P-value	<0.001

**Table 3:** Architect Versus UPLC Kappa Statistic

		UPLC Results			Total
		Deficiency	Insufficient	Sufficient	
Architect results	Deficiency	35	10	0	45
	Insufficient	1	2	2	5
	Sufficient	1	0	1	2
Total		37	12	3	52

Kappa, 0.252; p-value, 0.023 and %agreement, 73.1%

**Table 4:** COBAS E601 Versus UPLC Kappa Statistic

		UPLC Results			Total
		Deficiency	Insufficient	Sufficient	
Cobass Results	Deficiency	33	5	0	38
	Insufficient	3	6	1	10
	Sufficient	1	1	2	4
Total		37	12	3	52

Kappa, 0.509; p-value, <0.001 and %agreement, 78.8%

**Table 5:** Access 2 Versus UPLC Kappa Statistic

		UPLC Results			Total
		Deficiency	Insufficient	Sufficient	
Access results	Deficiency	35	9	0	44
	Insufficient	2	3	1	6
	Sufficient	0	0	2	2
Total		37	12	3	52

Kappa, 0.375; p-value, 0.002 and % agreement, 76.9%

**Table 6:** Clinical Guideline on Vitamin D status

Vitamin D Status	25 (OH) Vitamin D Concentration Range (ng/mL)	25 (OH) Vitamin D Concentration Range (nmol/L)
Deficient	< 20	< 50
Insufficient	20 to < 30	50 to < 75
Sufficient	30 -100	75 -250
Upper Safety Limit	>100	> 250

## Discussion

Our results revealed that the 3 immunoassays methods demonstrated acceptable performance. The results of these methods were comparable to those of UPLC based on Spearman Correlations. Based on the Bland-Altman difference plots, a mean bias of (-38.73%) was observed for the Abbott Architect, also a mean bias of (-4.22%) was observed for the Cobas, and a mean bias of (-12.34%) was observed for Access. 

Also, on doing further bias analysis on our results for the deficient samples only <50.0 nmol/L (< 30 ng/ml), it showed the best less bias with Cobas (-3.00%) followed by Access 2 (-7.75%) then comes the Architect (-37.32%). Thus, the three methods show less bias with lower concentrations of vitamin D especially on Cobas.

In agreement with our results, The bias of Architect was shown in a previous study by (Farrell et al., 2012) found a mean bias of 40.9% between the Abbott Architect and LC-MS/MS methods for whole study samples (N = 170) with concentrations ranging from 5.0 to 151.0 nmol/L and a mean bias of 104.5% for samples with concentrations <20 nmol/L (8 ng/ml) as assessed by LC-MS/MS.

Holmes et al., (2013) suggested that the Abbott Architect positive bias for samples at the lower end of the analytical measuring range is likely due to a standardization or calibration defect or a common, positive interfering substance that was not measured by LC-MS/MS. Also, Ong et al. also found significant positive bias for the Abbott Architect assay compared to LC-MS/MS (N = 200). Yet, clinically, 25(OH) D concentrations <50.0 nmol/L (30 ng/ml) indicate vitamin D deficiency; thus, inaccuracies at low concentrations have a limited impact on treatment decisions. 

In a study by Kocak et al., (2015) the Roche Cobas vitamin D total assay method showed a bias of (-14.1%), but in our study it is (-4.22%) observed for the Cobas e601.

Madenci et al., (2017) had examined Access 2 25 OH vitamin D assay by Bland–Altman analysis and a negative bias with the reference LC-MS/MS was seen (-19.2%).

Previous studies assumed that there are several explanations for the observed inter-method differences between the immunoassays and chromatography standard method. Bias may have resulted from different calibrator traceability. The Roche Cobas and Access2 vitamin D total assay has been standardized against chromatography which in turn has been standardized to the NIST standard. The Abbott Architect 25-OH vitamin D assay is traceable to the manufacturer’s internal standard (primary calibrator) against an absorbance of 264 nm. This may explain the lower bias in the case of Roche and Acess 2 method compared to Abbott method. 

In spite of the same Standard in both, the methodology of Access2 25(OH)D assay is a competitive chemiluminescence enzyme immune assay, while Cobas 25 (OH)D total assay is a competitive electrochemiluminescence immunoassay, explains bias between them (Madenci et al., in 2017).

Additionally, cross-reactivity may have occurred with other metabolites of 25 (OH) D. All immunoassays for 25 (OH) vitamin D show high cross-reactivity with 24, 25 (OH) 2D, which may be present in the serum at low concentrations, leading to higher results measured by immunoassays (Carter, 2012).

The Abbott assay manufacturer states that (112%) cross-reactivity occurs with 24, 25 (OH) 2D3, while Roche assay manufacturer claims to have (149%) cross-reactivity with 24, 25 (OH) 2D3. 

Also, matrix effects are known to occur in immunoassays and can lead to false high or low results. The most important type of matrix effect is any effect that occurs between the matrix in the calibrators and the patient samples (Wallace, et al., 2010). 

Abbott assay’s calibrators are composed of phosphate-buffered saline containing heat-inactivated horse serum and Roche assay’s calibrators contain human serum as a matrix. On the other hand, Access 2 has a multilevel serum calibrator.

Another factor may be the ability of an assay to separate 25 (OH) D from its binding protein. In chromatography methods, 25 (OH) D is separated from its binding protein by solvent extraction. However, in immunoassay methods, solvent extraction and chromatographic separation have been replaced by various blocking agents that displace 25 (OHD) from vitamin D binding protein (VDBP), which shows varying success. Although this simplified sample pre-treatment method enables the use of high sample throughput and automation, in case of incomplete extraction, false low 25 (OH) D concentrations may be obtained. Strong binding between the highly hydrophobic 25 (OH) D and VDBP creates competition with the capturing antibodies. VDBP must be inactivated or completely removed from the sample, as residual active VDBP at concentrations as low 2 ng/ml (0.5% of total VDBP) may interfere with the assay (Depreter et al., 2013). 

Results were then classified according to clinical status recommended of The Endocrine Society (Holick et al., 2012) shown in Table 6, we analyzed inter-rater agreement using the κ statistic and found strong to nearly perfect agreement in vitamin D status between the immunoassays and UPLC. 

Clinically, our results show higher agreement with cobas e601 with (p-value = <0.001 and %agreement = 78.8%) Followed by Access 2 (p-value = 0.002 and % agreement = 76.9%) then comes the Architect. Also, clinically 25 (OH) D concentrations <50.0 nmol/L (< 30 ng/ml) indicate vitamin D deficiency; thus, inaccuracies at low concentrations have a limited impact on treatment decisions. These results are consistent with the findings of previous studies (Madenci et al., 2017; Kocak et al., 2015).

According to CLSI EP09-A3 specifications, analysis by comparative and test methods should be performed within a time span consistent with analyte stability. For all analytes, the time span until analysis should not exceed 2 h for analysis for each method and, if possible, samples should be drawn on the day of analysis. In this study, 25(OH) D measurements by UPLC as well as measurements by immunoassay were performed within different time spans, but samples were kept at -20^o^C and used only once. A recent study by Colak et al. in 2013 reported that long-term frozen storage for short time does not affect serum vitamin D levels.

In conclusion, recently, a number of new automated assays have been introduced for 25-hydroxyvitamin D measurement. In this study we have investigated three immunological methods of its quantitation in correlation with a standard method UPLC. In general the immunological methods show good performance with varying bias. The most reliable method proved by our study is Cobas 6000 followed by the Access2 then comes Architect. Various causes of bias was discussed in our study. 
